# The Efficacy of Radiotherapy for Nasopharyngeal Carcinoma under Magnetic Resonance Imaging

**DOI:** 10.1155/2021/8280479

**Published:** 2021-07-30

**Authors:** Hao Zhang, Linlin Guo, Pengfei He, Zheng Chang

**Affiliations:** ^1^Department of Otorhinolaryngology-Head and Neck Surgery, The Second Hospital, Cheeloo College of Medicine, Shandong University, Jinan 250033, China; ^2^Department of Neurology, Zhangqiu District People's Hospital, Jinan 250200, China; ^3^Shandong Drug Addiction Monitoring and Treatment Institute, Jinan 250200, China; ^4^Intensive Care Unit, The Second Hospital, Cheeloo College of Medicine, Shandong University, Jinan 250033, China

## Abstract

This study aimed to analyze the application value of diffusion tensor imaging (DTI) in the diagnosis of nasopharyngeal carcinoma (NC) radiotherapy. In this study, 102 patients with NC were selected as the experimental group (EG), and 58 healthy people examined in hospital were included in a control group (CG). All subjects were required to be examined with routine magnetic resonance imaging (MRI) and DTI before and after the treatment. The fractional anisotropy (FA) of the patients in EG before and after treatment and the CG were recorded. The apparent diffusion coefficients (ADC) of patients in the two groups were measured and recorded before and after the treatment. The recovery rate and adverse events of the patients in EG were observed and recorded after the treatment. The results showed that the FA values of the right cerebellum and left parietal lobe (LPL) of patients after treatment in the EG were much higher than those before treatment and the CG (*P* < 0.05); the FA values of the right temporal lobe (RTL), right occipital lobe (ROL), and right parietal lobe (RPL) after treatment in the EG were obviously lower than those before the treatment and the CG (*P* < 0.05); the complete remission rate (CRR) of the EG after treatment was greatly higher than the partial remission rate (PRR) and disease stability rate (DSR) (*P* < 0.05), and the objective remission rate (ORR) and disease control rate (DCR) were higher than 90%, respectively. The ADC value of the EG before treatment was (0.752 ± 0.021) × 10^−3^ mm^2^/s, which was visibly lower than that after treatment ((1.365 ± 0.058) × 10^−3^ mm^2^/s) and that in the CG ((1.856 ± 0.079)) × 10^−3^ mm^2^/s), showing statistically obvious differences (*P* < 0.05). The incidence of anemia, oral reactions, hypertension, and gastrointestinal reaction in the EG after treatment was 61.46%, 45.35%, 47.28%, and 39.67%, respectively. In short, the FA value of DTI parameter could clearly indicate the changes in brain area characteristics of NC patients before and after treatment. The RTL, ROL, and RPL of NC patients were damaged after radiotherapy, and the FA value decreased observably, which may be related to brain edema and demyelination changes. The damage of white matter microstructure in each brain area further affected the cognitive function of the patient.

## 1. Introduction

Nasopharyngeal carcinoma (NC) refers to malignant tumors that occur on the top and side walls of the nasopharyngeal cavity. It is one of the high-incidence malignant tumors in China, and its incidence is the highest among the malignant tumors of the throat, ear, and nose [1, 2]. The common clinical symptoms are blood in the tears, nasal congestion, tinnitus, hearing loss, headache, facial numbness, diplopia, neck lumps, cranial nerve palsy, etc. In the later stage, there is a wasting syndrome characterized by weakness, progressive weight loss, fat loss, and muscle wasting [3, 4]. The occurrence of such disease is mainly related to infection, genetics, and environmental factors, such as heavy smoking, eating preserved food, and air pollution. The NC mainly was treated with radiotherapy clinically, including a comprehensive model of surgery, radiotherapy, and chemotherapy. Mostly, NC is moderately sensitive to radiotherapy, so radiotherapy is the first choice. However, radiation kills tumor cells and also damages surrounding normal tissue cells, eventually leading to radiation brain damage [5]. Radiation brain injury is a brain tissue radiation response syndrome, which may involve the temporal lobe, brainstem, basal ganglia, cervical spinal cord, and other parts, so it seriously affects the prognostic recovery and life quality of patients after radiotherapy [6]. Therefore, to improve the effect of radiotherapy and prevent the brain damage is very important for clinical treatment of NC.

At present, there is no uniform standard for the diagnosis of radiotherapy for patients with NC both in China and foreign countries. The traditional method is to judge whether there is radiation encephalopathy by the patients showing headache, learning and memory loss, and slow response, but the subjective interference factors are too strong and not accurate enough [7]. With the development of imaging, plain radiographs, computed tomography (CT), and magnetic resonance imaging (MRI) have been applied in the monitoring of brain tissue damage in early radiation therapy, but conventional plain radiographs, CT, and MRI cannot show the pathological changes of the brain tissue of patients in the acute reaction stage in time, and the diagnostic sensitivity is low. When the lesions such as white matter edema and encephalomalacia are shown, the radiation brain injury often has advanced to the late stage so that effective rescue cannot be realized [8, 9]. In recent years, some functional imaging and molecular imaging technologies have gradually developed on the basis of conventional MRI technology, mainly including magnetic resonance spectroscopy (MRS), diffusion weighted imaging (DWI), diffusion tensor imaging (DTI), perfusion-weighted imaging (PWI), and functional magnetic resonance imaging (fMRI) [10]. Among them, DTI is a new method of describing the structure of the brain, which can show the direction of nerve fiber bundles. It is also the only technology that can analyze the microstructure and morphology of white matter fibers in vivo [11, 12]. The apparent diffusion coefficient (ADC) can reflect the diffusion conditions inside the tissue, so as to evaluate the treatment effect to the tumor. Therefore, the DTI was intended to explore the brain tissue state of patients with NC after radiotherapy.

In summary, the application of DTI in the diagnosis of NC patients is a hot topic in current research. Based on this, 102 patients with NC who were admitted to the Second hospital of Shandong University were included in the experimental group (EG), and 58 healthy people in the imaging center during the same period were included in the control group (CG). All subjects were required to be examined with the routine MRI and DTI examinations before and after treatment. The FA value, ADC value, recovery rate (RR), and adverse events of the EG before and after treatment and the CG were compared and analyzed to comprehensively evaluate the application value of DTI in the diagnosis of NC radiotherapy.

## 2. Materials and Methods

### 2.1. Selection of Research Samples

In this study, 102 patients with NC, admitted to the hospital from January 31, 2018, to March 15, 2020, were selected as the EG (aged 20–67 years old). In addition, 58 healthy people with normal physical examination in the hospital during the same period were selected as the CG. The study had been approved by the Medical Ethics Committee of the hospital, and the patients and their families had understood the study and signed the informed consent forms.

The inclusion criteria were defined as follows: patients confirmed as NC by histopathology; patients receiving radiotherapy for the first time; patients in Han nationality; right-handed patients; patients with clear consciousness and ability to cooperate with the examination; and patients without metabolic diseases.

The exclusion criteria were defined as follows: patients who have taken relevant chemotherapy drugs; patients with psychiatric diseases; patients with intracerebral hemorrhage and clear vascular disease; patients with hypertension and diabetes; and patients with incomplete clinical data.

### 2.2. Radiotherapy

Radiation therapy was realized with the synergy + agility type medium energy linear accelerator (from Elekta, Swedish) for routine radiation. The dose for face and neck, preaural field, NC lesion, cervical lymph node, and skull base field was 38 Gy every 19 times, 32 Gy every 16 times, 70 Gy every 35 times, 64 Gy every 32 times, and 6 Gy every 3 times, respectively. Thus, dose for all locations could be deemed as 2 Gy each time.

Efficacy evaluation indicators: the radiotherapy effects were divided into CRR, PRR, DSR, ORR, and DCR. ORR=(CRR+PRR/total number of cases) × 100% and DCR=(CRR+PRR+DSR/total number of cases) × 100%.

### 2.3. Magnetic Resonance Imaging Scan of DTI

The 3.0 T MRI system produced by General Electric was adopted to collect resting state data on the subject's brain. The head coil was a standard 8-channel coil, and the enhanced scanning contrast agent was the gadopentetate meglumine injection (0.15 mmol/kg). During the scan, the subject was placed in a supine position, and the gap between the head and the 8-channel coil was filled with a foam pad to fix the head. A high-pressure syringe was employed to inject the 0.15 mmol/kg gadopentetate meglumine injection from the elbow vein. The subject was scanned from the top to the thoracic entrance, paying attention to be parallel and perpendicular to the third cervical vertebra. The conventional scanning parameters included time of repetition (TR) of 550 ms, time of echo (TE) of 35 ms, matrix of 521 × 521, layer thickness of 3.5 mm, layer spacing of 0.35 mm, and field of view of 25 × 25 cm.

DTI data collection was realized by using the single-shot plane echo sequence and parallel acquisition technology and was parallel to the front and back lines of the brain to obtain axial diffusion weighted images of the whole brain. The number of scanning layers was 40, and the scanning time was 300 seconds. The scanning parameters were set to that the TR was 11,000 ms, the TE was 85 ms, the matrix was 125 × 125, the layer thickness was 3.5 mm, the layer spacing was 0 mm, and the field of view was 25 × 25 cm.

### 2.4. Image Data Processing

The obtained images were sent to the workstation, and the DTI data were processed using the brain imaging data analysis tool FMRIB Software Library (FSL). Firstly, the eddy correct function in the FSL software was adopted for eddy current correction on the initial data, and then the brain region mask of each subject was obtained with the brain extraction tool (BET) to simplify the subsequent calculations. The DTIFit function was utilized to fuse the gradient magnetic field direction, the three-dimensional data, and the brain region mask data, so as to obtain a FA value.

### 2.5. Observation Indicators

The basic information (age, height, weight, and male and female ratio) were measured and recorded for subjects in both groups. ADC value [13] was used to evaluate subjects in the EG and CG before and after the treatment. The MRI images of patients were observed to determine the solid area and necrotic area of the lesion, outline the area of interest of the tumor, and then automatically obtain the ADC value of the perceptual area of interest using computer. The FA values of the right cerebellum, right temporal lobe (RTL), right occipital lobe (ROL), right parietal lobe (RPL), and left parietal lobe (LPL) of the EG before and after treatment and the CG were recorded and analyzed. The adverse events (anemia, oral reactions, hypertension, and gastrointestinal reactions) of patients in the EG were recorded before and after the treatment.

### 2.6. Statistical Methods

The data processing in this study was analyzed by SPSS 19.0 version statistical software, the measurement data were expressed as mean ± standard deviation $\left(\bar{x}\pm s\right)$, and the count data were expressed in percentage (%). The basic data of the EG and the CG were compared with the independent *t* test. The FA values of each brain area and ADC value were compared and analyzed with the one-way analysis of variance in the EG before and after the treatment and in the CG. The difference was statistically significant at *P* < 0.05.

## 3. Results

### 3.1. Comparison of Basic Data of the Two Groups of Subjects

**Figure 1:**
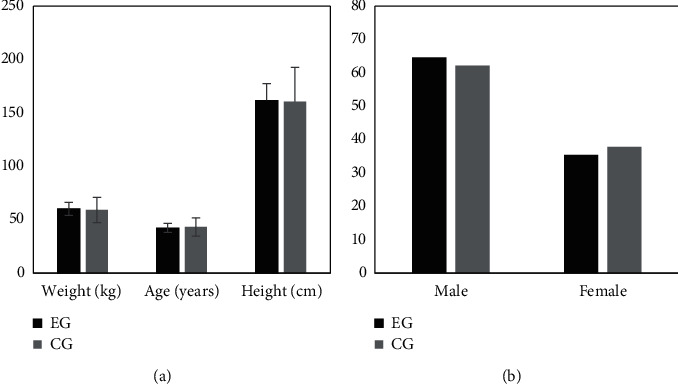
Comparison of basic data of the two groups of subjects. (a) The comparison results of the age, height, and weight of the two groups of subjects; and (b) the proportion of males and females of the two groups.

Figure 1 illustrates the comparison of basic data of the two groups of subjects. It reveals that the age of subjects was 42.17 ± 7.92 years old, the height was 161.33 ± 8.76 cm, the weight was 60.11 ± 11.45 kg, the proportion of males was 64.61%, and the proportion of females was 35.39% for patients in the EG. In addition, the age, height, weight, proportion of males, and proportion of females of the control subjects were 43.08 ± 9.22 years old, 160.57 ± 10.03 cm, 59.02 ± 9.07 kg, 62.18%, and 37.82%, respectively. Thus, the age, height, weight, proportion of males, and proportion of females in the EG were not statistically different from those in the CG (*P* > 0.05).

### 3.2. MRI Imaging Manifestations of Some Patients and People for Normal Control

**Figure 2:**
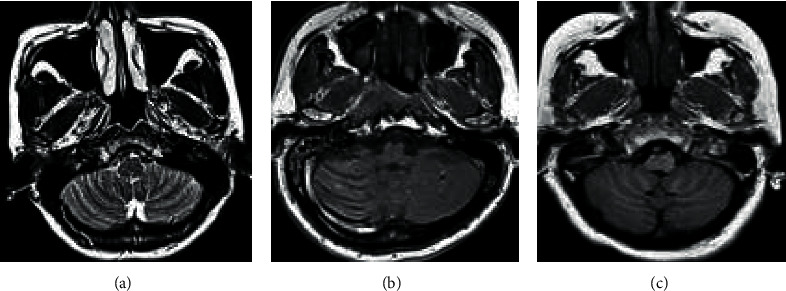
MRI imaging manifestations of some patients and healthy people. (a) An MRI image of a male patient with NC (aged 28 years old); (b) an MRI image of a female patient with NC (aged 35 years old); and (c) a normal nasopharyngeal MRI image of an adult (aged 25 years old).

Figure 2 shows the MRI images of some patients and healthy people.  Figure 2(a) shows the MRI findings of a male patient with NC. There were irregular soft tissue masses on the posterior wall and both sides of the nasopharynx, the signal was more and uniform, and liquefaction necrosis was rare; swollen lymph nodes in the neck were common; there were many enlarged lymph nodes with regular edges and uniform internal density or signal, and the enhanced scan was slightly enhanced.  Figure 2(b) shows the MRI findings of a female patient with NC. A soft tissue mass on the right posterior wall of the nasopharynx could be seen, which invaded the right parapharyngeal space, cervical sheath, and prevertebral muscles; the right posterior pharyngeal lymph nodes were enlarged, the pharyngeal recesses became shallow or disappeared, the nasopharyngeal contour changed, and the bilateral structure was asymmetrical.  Figure 2(c) shows the MRI findings of the nasopharynx of a normal adult. It discloses that the mucous membrane of the nasopharynx was thin, the surface was smooth and continuous, and the submucosal structure was clearly demarcated; the fossa was clearly displayed; and the signal in each sequence of MRI was normal.

### 3.3. FA Values of the Right Cerebellum, RTL, and ROL in the Patients before and after Treatment

**Figure 3:**
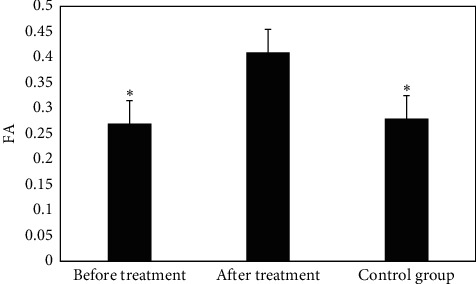
The FA values of the right cerebellum between the EG before and after the treatment and the CG. Note: *∗* indicates *P*< 0.05 in contrast to the value before treatment.

The FA values of the right cerebellum between the EG before and after the treatment and the CG were analyzed and are compared in Figure 3. FA value of the right cerebellum of the EG was 0.47 ± 0.12 and 0.32 ± 0.08 before and after the treatment, respectively; and the FA value of the right cerebellum in patients of the CG was 0.28 ± 0.07. It revealed that the FA value of the right cerebellum after treatment in the EG was higher obviously than that before treatment and the CG (*P* < 0.05).

**Figure 4:**
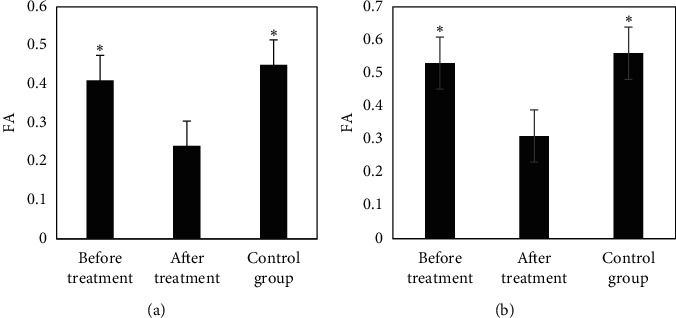
Comparison of FA values of RTL and ROL between the EG before and after treatment and the CG. (a) The FA value of the RTL; (b) the FA value of the ROL. Note: *∗* indicates *P* < 0.05 in contrast to the values before treatment.

Figure 4 compares FA values in the RTL and ROL of the EG before and after treatment to the CG. It reveals that the FA values of the RTL and ROL of the EG were 0.24 ± 0.07 and 0.33 ± 0.09 before the treatment, respectively; the FA values of the RTL and ROL of the EG were 0.41 ± 0.11 and 0.53 ± 0.12 after the treatment, respectively; and the FA values of the RTL and ROL of the patients in CG were 0.45 ± 0.12 and 0.58 ± 0.16, respectively. In addition, the FA values of the RTL and ROL after the treatment in the EG were dramatically lower than those before the treatment and those for patients in the CG (*P* < 0.05).

### 3.4. FA Values of LPL and RPL of the Patients between the EG before and after Treatment and the CG

**Figure 5:**
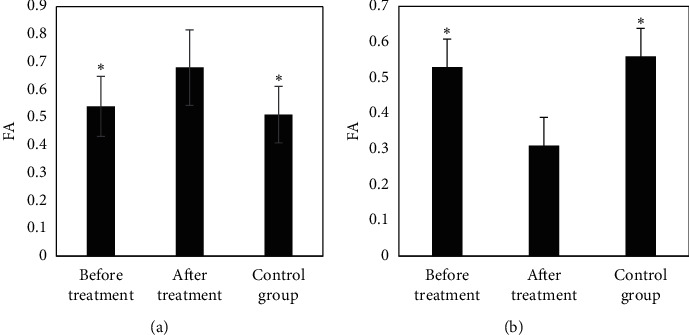
Comparison of FA values in the LPL and RPL of the EG before and after treatment to the CG. (a, b) FA values of the LPL and RPL, respectively. Note: *∗* indicates *P*< 0.05 in contrast to the values before treatment.

Figure 5 illustrates the comparison of FA values in the LPL and RPL of the EG before and after treatment to the CG. The results suggested that the FA values of the LPL and RPL of the EG were 0.68 ± 0.21 and 0.31 ± 0.09 before the treatment, respectively; the FA values of the RTL and ROL of the EG were 0.54 ± 0.13 and 0.53 ± 0.20 after the treatment, respectively; the FA values of the RTL and ROL of the CG were 0.51 ± 0.17 and 0.56 ± 0.14, respectively. In addition, the FA value of the LPL after treatment in the EG was much higher in contrast to that before treatment and the CG (*P* < 0.05); and the FA value of the RPL after treatment in the EG was much lower than that before treatment and the CG (*P* < 0.05).

### 3.5. Evaluation of the Cure Rate of Patients in the EG after Treatment

**Figure 6:**
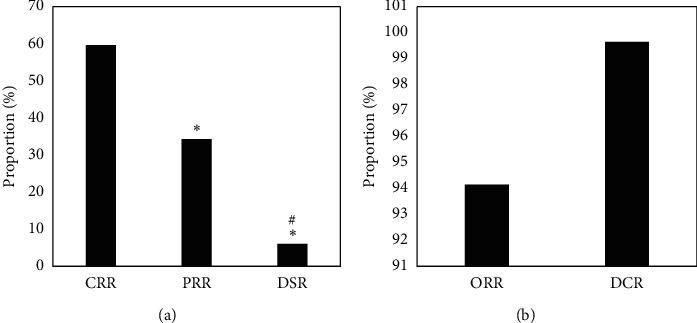
The evaluation results of the cure rate of patients in the EG. (a) The CRR, PRR, and DSR of patients after treatment; (b) The ORR and DCR of the patients after treatment. Note: *∗* indicates *P* < 0.05 in contrast to the CRR, and # suggests *P* < 0.05 in contrast to the PRR.

Figure 6 reveals the evaluation results of the cure rate of patients in the EG. It suggests that the CRR, PRR, DSR, ORR, and DCR of the EG after treatment were 59.63%, 34.28%, 6.09%, 94.15%, and 99.64%, respectively. Of which, the CRR after treatment in the EG was higher obviously in contrast to PRR and DSR (*P* < 0.05); the PRR after treatment in the EG was obviously increased in contrast to the DSR (*P* < 0.05); and the ORR and DCR of radiotherapy in the EG were higher, which were above 90%, respectively.

### 3.6. Comparison on ADC Values of Patients before and after the Treatment

**Figure 7:**
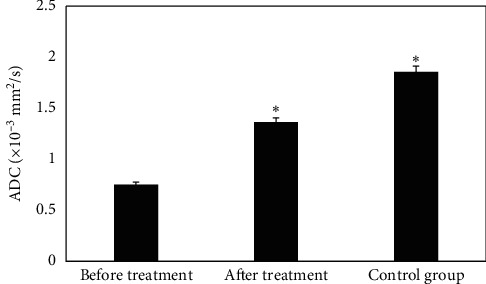
Comparison on ADC values of patients before and after the treatment. Note: *∗* suggests that the difference in ADC value was statistically obvious in contrast to the value before treatment (*P* < 0.05).

The ADC values of patients in the CG were compared before and after the treatment, and the results are illustrated in Figure 7. The ADC value of the EG before treatment was (0.752 ± 0.021) × 10^−3^ mm^2^/s, which was visibly lower than that after treatment ((1.365 ± 0.058) × 10^−3^ mm^2^/s) and that in the CG ((1.856 ± 0.079)) × 10^−3^ mm^2^/s), showing statistically obvious differences (*P* < 0.05).

### 3.7. Adverse Events after Treatment of Patients in the EG

**Figure 8:**
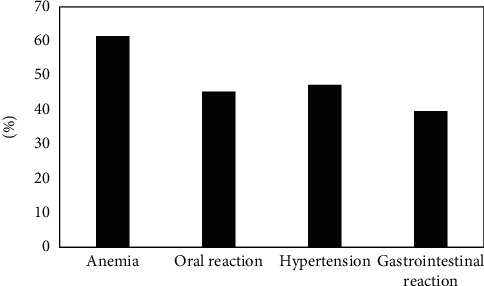
The adverse events of patients in the EG after treatment.

Figure 8 discloses the adverse events of patients in the EG after treatment. Figure 8 reveals that the incidence of anemia oral reactions, hypertension, and gastrointestinal reactions in the EG after treatment was 61.46%, 45.35%, 47.28%, and 39.67%, respectively.

## 4. Discussion

Due to the special location of NC (surrounded by the crucial organs), its anatomical structure and space are very narrow, so the clinical diagnosis and treatment were realized with radiotherapy. Although the effect is good, it is possible for large side effects, which mainly reflect in local facial skin changes, skin fibrosis, painful swallowing, reduced saliva, loss of smell and taste buds, and other manifestations. In addition, the occurrence is often related to the radiation dose, divided dose, and total dose [14]. As a new type of fMRI technology, DTI is a detection method that can evaluate the pathological information of living tissues. Therefore, 102 patients with NC were subjected to routine MRI and DTI scans before and after the treatment and 58 healthy people were subjected to the same scans. The results showed that the age, height, weight, proportion of male, and proportion of female in the EG were not statistically significant compared with the CG (*P* > 0.05), which indicated that the basic information of the two groups of subjects was consistent. The results of subsequent tests were comparable. After treatment, the FA values of the right cerebellum and LPL of the EG were higher observably than those before the treatment and the CG (*P* < 0.05), which was different from the decrease of FA values of cerebellar tissue analyzed by Qamar et al. [15]. The higher the value, the better the coherence and organization of the nerve fibers, reason of which may be that it is related to the change in the organization of nerve fiber bundles or the increased restriction of water molecule movement in the direction of nerve fibers [16, 17]. Therefore, it can be speculated that the increase in FA value is not entirely favorable.

After the treatment, the FA values of the RTL, ROL, and RPL in the EG were dramatically lower than those before the treatment and the CG (*P* < 0.05), which was similar to research results of King et al. [18], indicating that the white matter microstructure of the RTL, ROL, and RPL of NC patients was damaged after radiotherapy, and the FA value decreased hugely. The CRR of patients in the EG after the treatment was much higher than that of PRR and DSR (*P*< 0.05); and ORR and DCR were above 90%, indicating that radiotherapy was more effective in patients with NC. The ADC value of the EG before treatment was (0.752 ± 0.021) × 10^−3^ mm^2^/s, which was visibly lower than that after treatment ((1.365 ± 0.058) × 10^−3^ mm^2^/s) and that in the CG ((1.856 ± 0.079)) × 10^−3^ mm^2^/s), showing statistically obvious differences (*P* < 0.05). Such results were similar to the findings of Mao et al. [19]. The rising trend of ADC value is closely related to tumor shrinkage and remission. After the treatment, the incidence of anemia, oral reactions, hypertension, and gastrointestinal reactions in the EG was 61.46%, 45.35%, 47.28%, and 39.67%, respectively. Thus, the adverse events of radiotherapy were still relatively severe, which may be related to the dose. Therefore, related preventive measures had to be adopted in clinical treatment in terms of the postoperative side effects [20].

## 5. Conclusion

In this study, 102 patients with NC who were admitted to the hospital were selected as the EG and 58 healthy people who underwent routine physical examination in the imaging center of the hospital during the same period were selected as the CG. All subjects were required to be examined with the routine MRI and DTI examinations before and after treatment. The results showed that the RTL, ROL, and RPL of NC patients were damaged after radiotherapy, and the FA value decreased significantly, which may be related to changes in cerebral edema and demyelination. The damage of white matter microstructure in each brain area further affected the cognitive function of the patient. However, there are still some shortcomings for this study. It was difficult to obtain the brain tissue samples for pathological diagnosis for NC patients with normal MRI findings, and the impact of chemotherapy drugs had not been analyzed. In future, it will increase the size of selected sample to eliminate the drug difference and discuss the effect of radiotherapy for NC. In conclusion, the results provided an experimental help for the application of DTI in radiotherapy for NC.

## Data Availability

The data used to support the findings of this study are available from the corresponding author upon request.

## References

[1] Song C., Cheng P., Cheng J. (2020). Differential diagnosis of nasopharyngeal carcinoma and nasopharyngeal lymphoma based on DCE-MRI and RESOLVE-DWI. *European Radiology*.

[2] Zhang B., Tian J., Dong D. (2017). Radiomics features of multiparametric MRI as novel prognostic factors in advanced nasopharyngeal carcinoma. *Clinical Cancer Research*.

[3] Lin L., Dou Q., Jin Y.-M. (2019). Deep learning for automated contouring of primary tumor volumes by MRI for nasopharyngeal carcinoma. *Radiology*.

[4] Chan S.-C., Yeh C.-H., Yen T.-C. (2018). Clinical utility of simultaneous whole-body 18F-FDG PET/MRI as a single-step imaging modality in the staging of primary nasopharyngeal carcinoma. *European Journal of Nuclear Medicine and Molecular Imaging*.

[5] King A. D., Woo J. K. S., Ai Q. Y. (2019). Complementary roles of MRI and endoscopic examination in the early detection of nasopharyngeal carcinoma. *Annals of Oncology*.

[6] Ai Q.-Y., King A. D., Chan J. S. M. (2019). Distinguishing early-stage nasopharyngeal carcinoma from benign hyperplasia using intravoxel incoherent motion diffusion-weighted MRI. *European Radiology*.

[7] Mao J., Fang J., Duan X. (2019). Predictive value of pretreatment MRI texture analysis in patients with primary nasopharyngeal carcinoma. *European Radiology*.

[8] Zhao L., Gong J., Xi Y. (2020). MRI-based radiomics nomogram may predict the response to induction chemotherapy and survival in locally advanced nasopharyngeal carcinoma. *European Radiology*.

[9] Xu Y., Rong X., Hu W. (2018). Bevacizumab monotherapy reduces radiation-induced brain necrosis in nasopharyngeal carcinoma patients: a randomized controlled trial. *International Journal of Radiation Oncology∗Biology∗Physics*.

[10] Li Z., Li Y., Li N., Shen L. (2019). Positron emission tomography/computed tomography outperforms MRI in the diagnosis of local recurrence and residue of nasopharyngeal carcinoma: an update evidence from 44 studies. *Cancer Medicine*.

[11] Ming X., Oei R. W., Zhai R. (2019). MRI-based radiomics signature is a quantitative prognostic biomarker for nasopharyngeal carcinoma. *Scientific Reports*.

[12] Zhang L.-L., Huang M.-Y., Li Y. (2019). Pretreatment MRI radiomics analysis allows for reliable prediction of local recurrence in non-metastatic T4 nasopharyngeal carcinoma. *EBioMedicine*.

[13] Wang M.-L., Wei X.-E., Yu M.-M., Li W.-B. (2017). Value of contrast-enhanced MRI in the differentiation between nasopharyngeal lymphoid hyperplasia and T1 stage nasopharyngeal carcinoma. *La Radiologia Medica*.

[14] Feng Y., Cao C., Hu Q., Chen X. (2019). Grading of MRI-detected skull-base invasion in nasopharyngeal carcinoma with skull-base invasion after intensity-modulated radiotherapy. *Radiation Oncology*.

[15] Qamar S., King A. D., Ai Q.-Y. (2019). Amide proton transfer MRI detects early changes in nasopharyngeal carcinoma: providing a potential imaging marker for treatment response. *European Archives of Oto-Rhino-Laryngology*.

[16] Yan X. J., Fang S., Huang G. W. (2018). Clinical study of nasopharyngeal masses with suspicion of nasopharyngeal carcinoma in adult patients. *Zhonghua Er Bi Yan Hou Tou Jing Wai Ke Za Zhi*.

[17] Ai Q.-Y., King A. D., Mo F. K. F. (2018). Staging nodal metastases in nasopharyngeal carcinoma: which method should be used to measure nodal dimension on MRI?. *Clinical Radiology*.

[18] King A. D., Vlantis A. C., Yuen T. W. C. (2015). Detection of nasopharyngeal carcinoma by MR imaging: diagnostic accuracy of MRI compared with endoscopy and endoscopic biopsy based on long-term follow-up. *American Journal of Neuroradiology*.

[19] Mao J., Shen J., Yang Q. (2016). Intravoxel incoherent motion MRI in differentiation between recurrent carcinoma and postchemoradiation fibrosis of the skull base in patients with nasopharyngeal carcinoma. *Journal of Magnetic Resonance Imaging*.

[20] Zhuo E.-H., Zhang W.-J., Li H.-J. (2019). Radiomics on multi-modalities MR sequences can subtype patients with non-metastatic nasopharyngeal carcinoma (NPC) into distinct survival subgroups. *European Radiology*.

